# Frederick Brittan

**Published:** 1891-03

**Authors:** 


### Obituary.

### FREDERICK BRITTAN, B.A., M.D., M.R.C.S.

The recent death of Dr. Brittan reminds us that the present Bristol Medico-Chirurgical Society owes much to him, because the support he gave it at its formation, and the philosophic character of the inaugural address which he delivered as its first President, indicated the lines upon which it should work, and went far to ensure for it the success which it has attained.

Frederick Brittan was born in 1823. His father was Meshach Brittan, a well-known solicitor of Bristol, who had several other sons, two of whom survive. He received his general education at private schools in his native town, being for a time in the junior department of the old Bristol College, where so many of the older generation here were educated; very many of whom have already passed away. For a short time he went to a school kept by the Rev. George Southwell, at 10 Richmond Hill; and the writer of this notice, who, with one of his brothers, was then at the head of the school, well remembers the advent of a couple of round-faced, chubby little boys, who in process of time developed into Dr. Brittan and his brother Alfred, now a solicitor in this city.

Thence they went early to Dublin, where they finished their general education at Trinity College, taking their degrees of B.A. in 1842, Frederick Brittan taking the additional degree of M.B. in the same year.

Being then only in his twentieth year, he came back to Bristol, and took a year's dressership under Mr. Lowe, one of the surgeons to the Infirmary; but he never attended the lectures at the Bristol Medical School. He subsequently went to Paris, and was a student in the hospitals there for another year. In 1844 he passed the

College of Surgeons; and in 1845, after going back to Dublin to take the M.D. degree, settled in Clifton.

Mr. Brittan, as he was then called, according to the commendable custom at that time prevailing of giving the title of Dr. to those only who professed to practise as physicians, after starting as surgeon, with the orthodox brass-plate on his door, began his work by publishing a translation of Malgaigne's *Operative Surgery*, which was well received; for Malgaigne was at that time one of the best known among the active Parisian surgeons.

In 1848 he joined the Bristol Medical School, where he lectured on various subjects, altogether for twenty years; being first chosen for the Chair of General Anatomy and Physiology. He was a popular and successful lecturer, and it will be of interest to have a record of his official connection with the School. He undertook the whole course of General Anatomy and Physiology from October, 1848, to April, 1853, at a time when the course meant a lecture six days in the week for six months, or one hundred and twenty lectures in the Winter Session; and at the same time he gave a summer course on Vegetable Physiology, as part of the Botanical Lectures—a subject imperative on all students in those days. He gave up the latter in 1854, and was succeeded by Robert Etheridge, whose name is well known in the scientific world in connection with geological and botanical subjects. In 1853–54 he gave half the course of Physiology and half of that on Descriptive and Surgical Anatomy, as a colleague of Mr. Prichard, who had taken half the Surgical lectures; and for the two following years as a colleague of Mr. Leonard. In 1855 he gave half the course of Physiology and half the course on Medicine with Dr. Stanton; and then he had all the lectures on Medicine until 1868, when he shared the course with the late Dr. Martyn of the General Hospital, and then resigned, being succeeded by Dr. Edward Long Fox.

In the year 1856, Mr. Brittan, who to this date had been practising as a surgeon, and was looked upon as one of the future candidates for the surgeoncy to the Infirmary should a vacancy occur, changed suddenly into Dr. Brittan, and was elected one of the Physicians to the Infirmary, under the following peculiar circumstances. When it was known that the post would soon become vacant by the resignation of Dr. James Bernard, and that an unpopular candidate was coming forward and was likely to be successful, the faculty sent a deputation to offer Mr. Brittan their unanimous support if he would come forward and stand for the post. To this he assented, and was accordingly elected; and he held the office until 1873, when he resigned and was made Consulting Physician.

This notice would be incomplete without some reference to the active part taken by Dr. Brittan at the time of the severe outbreak of cholera in Bristol, in 1849. The Medico-Chirurgical Society of the time determined to investigate the subject as thoroughly as possible, and formed pathological, microscopical, and chemical sub-committees of its members to make various researches and examinations of specimens, and to report the results. The Microscopical Committee were Dr. James Bernard, Dr. William Budd, Mr. Neild (one of the surgeons to the General Hospital), Mr. Brittan, Mr.

Joseph Swayne, and Mr. Prichard. Specimens of the excreta (rice-water evacuations, etc.) were obtained and carefully examined by all, but more particularly by Messrs. Brittan and Swayne, who both reported the discovery of certain cells, compound and simple, which they called cholera bodies or cells, and which they searched for in vain in corresponding secretions that were not choleraic. The same bodies were found in the substance scraped from the mucous membrane of the intestine at the *post mortem* examinations of cholera subjects, and Dr. Budd found some very similar organisms in specimens of drinking-water obtained from infected districts. Then Mr. Brittan, at the suggestion of Dr. James Bernard, condensed, with some trouble and much perseverance, a drachm or two of liquid from the air of a house from which five patients had been recently removed to the Cholera Hospital, two of whom died, and also from a cell in Bridewell, in which a man had just died, and in this fluid similar, but smaller, bodies were found.

The report of the Sub-Committee was published in *The Provincial Medical and Surgical Journal*, and diagrams and copious notices were inserted in that journal and in the *Lancet* and *Medical Times and Gazette;* and the matter was discussed largely in the London daily papers, to some of which letters and long articles had been sent. Dr. Budd, in a letter to the London *Times*, stated his belief in the germ theory in the propagation of cholera.[1] There was at that time a good deal of friction between some members of the committee, as sometimes happens in such cases even among members of our profession; but it was transient.

Then appeared the authoritative dictum, the bluntly-expressed manifesto of the Cholera Sub-Committee of the Royal College of Physicians (Dr. Baly and Dr. Gull), flatly contradicting the accuracy of the observations and deductions. They said that "bodies having the form of so-called cholera fungi are not to be detected in the air nor in the water;" that a "large number of these bodies have been traced to articles of medicine or food;" that "the most remarkable forms are to be discovered in the intestinal evacuations of persons labouring under diseases totally different from cholera;" and, as a conclusion to be drawn from their researches, that "the whole theory of the disease which has recently been propounded is erroneous, as far as it is based on the existence of the bodies in question." This led, of course, to some controversial letters in the journals; but the *ex cathedrâ* report of the College was accepted by the majority of the profession and also by the public, who believed then, as now, that medical and surgical skill is a good deal restricted to a circle with a limited radius from the General Post Office in London.

One result of the publicity given to this subject was an offer made by Government to Mr. Brittan to undertake the organisation and superintendence of the medical care of Taunton, where a sharp outbreak of cholera had occurred; and as it was early in his practice, which did not increase very rapidly, and the offer was five guineas a day and expenses, he had no hesitation about accepting it.

He was elected president of the Bath and Bristol Branch of the

1 Dr. Budd, soon after this period, expressed his opinion that phthisis tubercularis is a zymotic disease.

British Medical Association in 1865, in succession to Dr. Falconer of Bath, and in his turn was succeeded by Mr. John Bartrum; and when the Bristol Medico-Chirurgical Society was resuscitated, in 1874, he was the first new president; his address and the discussion touching chiefly on the subject of contagion and the germ theory.

Dr. Brittan was careful and painstaking in the examination of his patients, and a help in the diagnosis of a difficult case. He had a considerable practice as consulting physician, and many patients of his own whom he generally attended; but he always managed to find time for a little shooting or fishing or painting, when he had a mind for a holiday. He was a private in the Bristol Volunteer Rifles when they were first established, and did some successful prize shooting.

He married very early in his career, and left one son, a solicitor, in Bristol; and after the death of his wife he retired from practice altogether in 1882, and went to live near Glandovey, Cardiganshire. He married again, leaving by his death a widow and two young children. His health had been somewhat precarious for some time, for he suffered from albuminuria and hypertrophy of the heart; and his death, which occurred in London, where he was temporarily staying, was very sudden, on the fifteenth day of February of this year, on the thirty-third anniversary of his election as Physician to the Bristol Royal Infirmary.

